# Quantum simulation of the Hubbard model with dopant atoms in silicon

**DOI:** 10.1038/ncomms11342

**Published:** 2016-04-20

**Authors:** J. Salfi, J. A. Mol, R. Rahman, G. Klimeck, M. Y. Simmons, L. C. L. Hollenberg, S. Rogge

**Affiliations:** 1 Centre for Quantum Computation and Communication Technology, School of Physics, The University of New South Wales, Sydney, New South Wales 2052, Australia; 2 Department of Electrical Engineering, Purdue University, West Lafayette, Indiana 47906, USA; 3 Centre for Quantum Computation and Communication Technology, School of Physics, University of Melbourne, Parkville, Victoria 3010, Australia

## Abstract

In quantum simulation, many-body phenomena are probed in controllable quantum systems. Recently, simulation of Bose–Hubbard Hamiltonians using cold atoms revealed previously hidden local correlations. However, fermionic many-body Hubbard phenomena such as unconventional superconductivity and spin liquids are more difficult to simulate using cold atoms. To date the required single-site measurements and cooling remain problematic, while only ensemble measurements have been achieved. Here we simulate a two-site Hubbard Hamiltonian at low effective temperatures with single-site resolution using subsurface dopants in silicon. We measure quasi-particle tunnelling maps of spin-resolved states with atomic resolution, finding interference processes from which the entanglement entropy and Hubbard interactions are quantified. Entanglement, determined by spin and orbital degrees of freedom, increases with increasing valence bond length. We find separation-tunable Hubbard interaction strengths that are suitable for simulating strongly correlated phenomena in larger arrays of dopants, establishing dopants as a platform for quantum simulation of the Hubbard model.

## Abstract (web summary)

The goal of quantum simulation is to probe many-body phenomena in controlled systems, but Fermi-Hubbard phenomena are typically hard to simulate in cold atomic. Here, the authors simulate them with subsurface dopants in silicon, achieving a low effective temperature and reading out spin states with STM.

## Graphical abstract

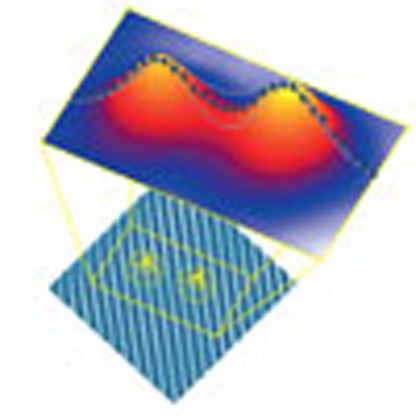

Quantum simulation offers a means to probe many-body physics that cannot be simulated efficiently by classical computers, using controllable quantum systems to physically realize a desired many-body Hamiltonian^1^,^2^,^3^. In the analogue approach to quantum simulation exemplified by cold atoms in optical lattices^4^,^5^, the simulator's Hamiltonian maps to the desired Hamiltonian. Compared to digital quantum simulation, realized via complex sequences of gate operations^6^,^7^, analogue quantum simulation is usually carried out with simpler building blocks. For example, the Heisenberg and Hubbard Hamiltonians of great interest in many-body physics are directly synthesized by cold atoms in optical lattices^2^,^3^. Although of immense interest and proposed long ago^8^, analogue simulation of fermionic Hubbard systems has proven to be very challenging^2^,^3^. The anticipated regime of the intensely debated spin liquid, unconventional superconductivity and pseudogap^9^,^10^,^11^ has yet to be accessed even for cold atoms. Here, the required low temperature *T*<*t*/30 is problematic due to the weak tunnel coupling *t* of cold atoms^5^,^12^. Moreover, experimentally resolving individual lattice sites, crucial elsewhere in Bose–Hubbard simulation^4^, remains very challenging in quantum simulation of the Hubbard model^5^.

Here, we perform atomic resolution measurements resolving spin–spin interactions of individual dopants, realizing an analogue quantum simulation of a two-site Hubbard system. We demonstrate the much desired combination of low effective temperatures, single-site spatial resolution, and non-perturbative interaction strengths of great importance in condensed matter^9^,^10^,^11^. The dopants' physical Hamiltonian 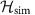, determined at the time of fabrication^3^, maps to an effective Hubbard Hamiltonian 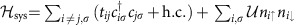, where 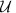 is the on-site Coulomb repulsion, 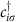 (*c*_*iσ*_) creates (destroys) a fermion at lattice site *i* with spin *σ*, 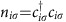 is the number operator, and h.c. is the Hermitian conjugate. Here, it is desirable to achieve non-perturbative (intermediate) interaction strengths 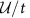 associated with quantum fluctuations and emergent phenomena^9^,^10^,^11^, that is, beyond perturbative Heisenberg interactions (large 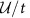) realized in photon-based^13^ and ion-based^14^ simulations, and magnetic ions on metal surfaces^15^. We focus on the system ground state, prepared by relaxation on cooling^3^, rather than system dynamics.

Because the states of our artificial Hubbard system are coupled and interacting, tunnelling spectroscopy locally probes the spectral function. The spectral function is of key interest in many-body physics because it provides rich information on interactions^16^,^17^, and is highly sought after in future ‘cold-atom tunnelling microscope' experiments^18^. For our few-body system, the local spectral function describes the quasi-particle wavefunction (QPWF)^19^,^20^,^21^,^22^ and the discrete coupled-spin spectrum of the dopants. We find that interference of atomic orbitals directly contained in the QPWF allows us to quantify the electron–electron correlations and the entanglement entropy. The entanglement entropy is a fundamental concept for correlated many-body phases^23^,^24^,^25^,^26^ that has thus far evaded measurement for fermions. In the counterintuitive regime of our experiments, entanglement entropy increases as the valence bond is stretched, as Coulomb interactions overcome quantum tunnelling. In our system, the entanglement entropy is directly related to the Hubbard interactions 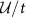, and we find that 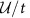 is tunable with dopant separation, increasing from 4→14 for *d*/*a*_B_=2.2→3.7, where *a*_B_=1.3 nm is the effective Bohr radius. This range, of interest to simulate unconventional superconductivity and spin liquids^9^,^10^,^11^, is realized here due to the large Bohr radii of the hydrogenic states. The semiconductor host allows for electrostatic control of the chemical potential^27^,^28^, desirable to dynamically control filling factor^9^,^11^ but not possible for ions on metal surfaces^15^.

## Results

### Spectroscopy of coupled-spin system

Subsurface boron acceptors in silicon were identified at 4.2 K as individual protrusions^29^,^30^ (density ∼10^11^ cm^−2^) in constant current images due to resonant tunnelling at a sample bias *U*=+1.6 V, and due to the acceptor ion's influence on the valence density of states at *U*=−1.5 V. The sample was prepared by ultra-high vacuum flash annealing at 1,200 °C and hydrogen termination. The observed subsurface acceptors had typical depths^29^,^30^ <3 nm, and correspondingly, a volume density >25 times less than the bulk doping 8 × 10^18^ cm^−3^. Pairs of nearby acceptors with *d*≲5 nm were also found, with a smaller density ∼10^9^ cm^−2^.

**Figure 1:**
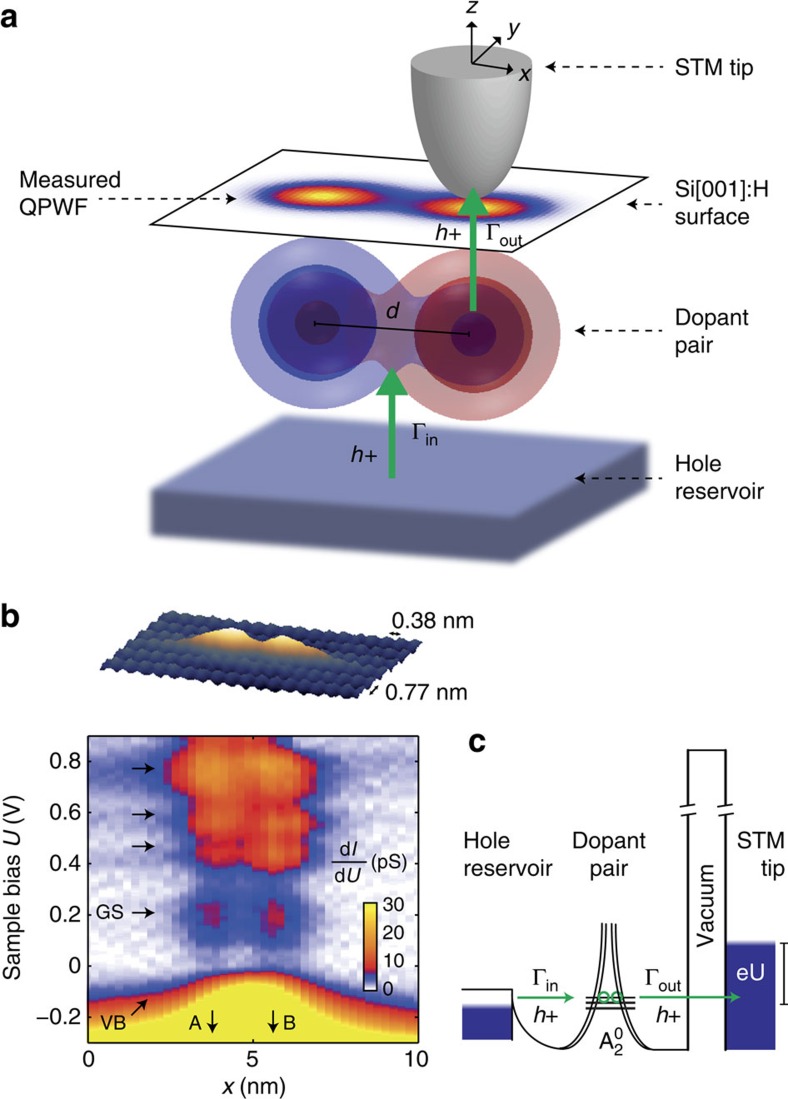
Spatially resolving coupled-spin states. (**a**) Atomic resolution single-hole tunnelling probes the interacting states of coupled acceptor dopants (Γ_out_=tunnel rate to tip, 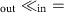
tunnel rate from reservoir). The inter-acceptor coupling *t* obeys 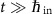. d*I*/d*U* measures the interacting states' QPWF, which contains interference processes connnected to two-body wavefunction amplitudes, the entanglement entropy and effective Hubbard interactions. (**b**) Acceptor pair (double-protrusion) in topography at *U*=+1.8 V and *I*=300 pA (top), and spectrally and spatially resolved d*I*/d*U* taken at a bias *U*=+2.0 V, where topography is flat apart from atomic corrugation (bottom). VB, 2-hole ground state and 2-hole excited states are indicated. (**c**) Effective energy diagram of sequential hole tunnelling through 2-hole ground and excited state of coupled acceptors.

The spectrum and spatial tunnelling probability of the coupled acceptors were investigated at *T*=4.2 K via single-hole tunnelling from a reservoir in the substrate to the dopant pair, to the tip^29^,^30^ (Fig. 1a). For the dopant pair in Fig. 1b (top), d*I*/d*U* measured along the inter-dopant axis (Fig. 1b, bottom) contains a peaks for each state entering the bias window, at *U*≈0.2, 0.45, 0.55 and 0.8 V. Consistent with our single-acceptor^29^ and single-donor^31^ measurements near flat-band bias conditions, the bias for each peak in the spectrum (Fig. 1b, bottom) is independent of tip position. This rules out distortion of our quantum state images by inhomogenous tip-induced potentials^32^ observed in other multi-dopant systems^33^. These results can be attributed to weak electrostatic control by the tip (Fig. 1c) and the states' proximity to flat-band^29^,^30^,^31^, though a large tip radius may also play a role.

The spectral and spatially resolved measurements (Fig. 1b) directly demonstrate that the holes are interacting, as follows. First, two peaks centred on dopant ions A or B are resolved in real space (Fig. 1b). Second, energy differences between the peaks resolved in real space are smaller than the ∼350 μeV thermal resolution. However, for orbitals at the same energy to not interact, their overlap must vanish. Since the measured orbitals have a strong overlap, the sites are tunnel coupled, irrespective of the details of the tunnelling current profile. The number of states observed, their energy differences, and their energies relative to the Fermi energy confirm that the observed states are two-hole states (Supplementary Figs 1 and 2).

### Correlations and entanglement from Hubbard interactions

**Figure 2:**
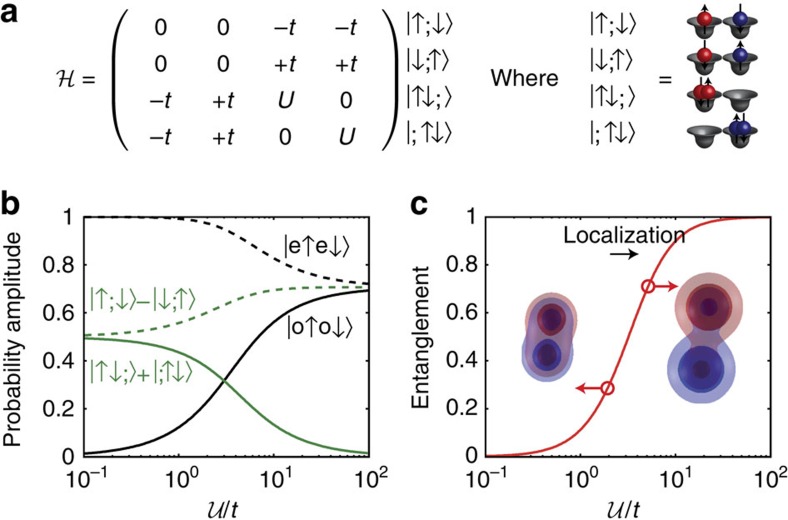
Hubbard interactions and entanglement entropy. (**a**) Two-site Hubbard Hamiltonian in the subspace of the ground state, with tunnel coupling *t* hybridizing singly- and doubly-occupied configurations, for sites *A* (red orbital) and *B* (blue orbital). (**b**) Dependence of probability amplitudes on interactions 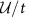: *γ*_c_ (green dashed) and *γ*_i_ (green solid) for configurations 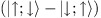 and 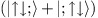, and *γ*_ee_ and *γ*_oo_ for 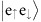 and 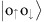, respectively. (**c**) Entanglement entropy 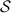 increases with increasing Hubbard interactions 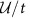. This occurs because of localization of red and blue orbitals associated with spins in the singlet, as illustrated in the insets.

The ground state of a Hubbard model with non-perturbative interactions is governed by 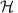 in Fig. 2a in the subspace of 
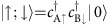
, 
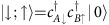
, 
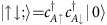
 and 
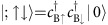
, where 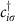 creates a localized electron on site *i*∈{A, B} with spin 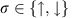, and 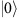 is the vacuum state. The ground state is a superposition 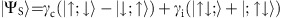, where *γ*_c_ (*γ*_i_) is the probability amplitude for a covalent (ionic) configuration (Fig. 2b). Rewriting the state in a basis of even and odd orbitals, 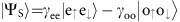, where *γ*_ee_ (*γ*_oo_) is the probability amplitude of the ‘even/even' (‘odd/odd') configuration.

In limit of small tunnel couplings (large 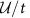, Fig. 2b) the Hubbard system may be described by perturbative Heisenberg spin interactions. For vanishing 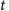, the ground state is a Heitler–London singlet of localized spins, 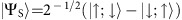, with no contributions from 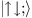 and 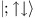. Due to vanishing wavefunction overlap the electrons can be associated with sites A and B (they are distinguishable^23^,^34^,^35^), and the spin at site A depends on the spin at site B as for a maximally entangled Bell state. In the limit of vanishing interactions (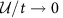, Fig. 2b) corresponding to a tight-binding approximation, the spins delocalize and 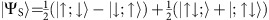. In a molecular orbital (MO) basis, the ground state is 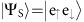, which is a single Slater determinant. Although this state is a singlet (one spin up, one spin down) due to fundamental indistinguishability, the electrons can be ascribed independent properties because they occupy the same orbital, and the state is uncorrelated^23^,^34^,^35^.

For intermediate 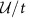, where tunnelling and Coulomb interactions compete non-perturbatively^2^,^3^,^9^,^11^, tunnelling hybridizes the doubly-occupied configurations 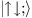 and 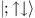 into the ground state, such that the particles lose their individual identities. Here, the von Neumann entanglement entropy quantifies genuine entanglement (inter-dependency of properties), distinguishing it from exchange-correlations due to indistinguishability^23^,^26^,^35^. Employing the convention^36^ 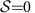 (1) for zero (maximal) entanglement, 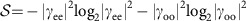 increases as 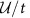 increases and coherent localization occurs (Fig. 2c), saturating at value of 1.

**Figure 3:**
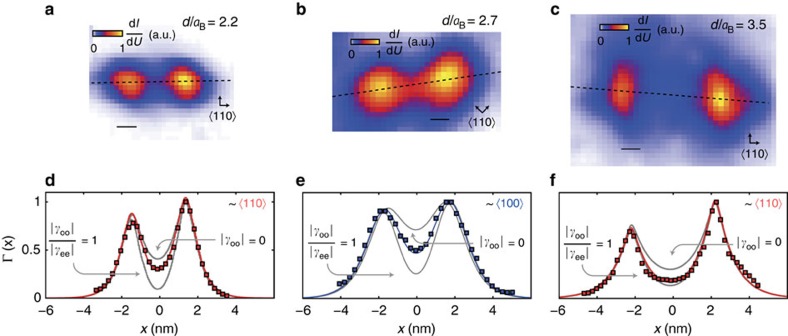
Resolving interference processes in quasi-particle wave function. (**a**) Experimentally measured, normalized tunnelling probability Γ∝d*I*/d*U* to tip, for *d*=2.2*a*_B_ ground state. Arrows denote 110 crystal directions. (**b**) Same as (**a**), for *d*=2.7*a*_B_. (**c**) Same as (**a**) for *d*=3.5*a*_B_. (**d**) Normalized experimental line profile (coloured squares) of Γ(*x*) for *d*=2.2*a*_B_ and least-squares fit (coloured line) to QPWF correlated singlet model. Lower and upper grey lines are line profiles of maximally and minimally correlated states, obtained from least square fits. The maximally correlated state deviates from the mean of |*ϕ*_*e*_(**r**)|^2^ and |*ϕ*_*o*_(**r**)|^2^ because of the different normalization coefficients of even and odd linear combinations. (**e**) Same as (**d**) for *d*=2.7*a*_B_. (**f**). Same as (**d**) for *d*=3.5*a*_B_. Scale: 1 nm.

We now discuss the spatial tunnelling maps of the two-hole ground states for different inter-acceptor distances. Obtained by integrating the lowest voltage d*I*/d*U* peak, the maps are shown in Fig. 3a–c for distances *d*/*a*_B_=2.2, 2.7 and 3.5 (*a*_B_=1.3 nm) having orientations ±2° from 〈110〉, 8±2° from 〈100〉 and 3±2° from 〈110〉, respectively. The multi-nm spatial extent of the states reflects the extended wave-like nature of the acceptor-bound holes, owing to their shallow energy levels, which contrasts Mn ions on GaAs surfaces^37^, magnetic ions on metals^15^, and Si(001):H dangling bonds^38^. Consequently, their envelopes are amenable to effective-mass analysis with lattice frequencies filtered out^19^,^20^,^28^,^39^. Consistent with measurements of single acceptors at similar depths on resonance at flatband^29^,^30^, the states have predominantly *s*-like envelopes with slight extension along [110] directions, as expected when symmetry is not strongly perturbed by the surface. Depths of the *d*/*a*_B_=2.7 and *d*/*a*_B_=3.5 pairs were estimated to be ∼0.9 nm, and for *d*/*a*_B_=2.2, ∼0.6 nm (see Supplementary Fig. 3).

We employed full-configuration interaction calculations of the singlet ground state 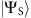 to confirm that Coulomb correlations of coupled acceptors influence the ground state in a way that mimics the *S*=1/2 Hubbard model. In particular, for *d*/*a*_B_∼2, 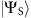 is predominantly composed of 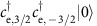, a singlet of two even ±‘3/2' spin MOs. With increasing *d*, interactions enhance the probability amplitude of the 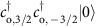 singlet with two odd orbitals, analogous to the Hubbard Hamiltonian (Fig. 2b). The spins ±‘3/2' are predominantly composed of 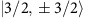 valence band (VB) Bloch states. In particular, the low-lying ±‘1/2' spin excitations of each acceptor^30^, which are predominantly composed of 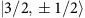 Bloch states, do not qualitatively change the description. We also note that for *d*/*a*_B_⪞2, the MOs are essentially linear combinations atomic orbitals having the effective Bohr radii of single acceptors.

Single-hole tunnelling transport through our coupled-dopant system locally probes the spectral QPWF^19^,^20^,^21^. When 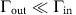 (Fig. 1a), the single-hole tunnelling rate is essentially governed by Γ_out_, the tunnel-out rate^31^. In the present case, single-hole tunnelling from the two-hole system to a single-hole final state 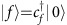 (Fig. 1) contributes 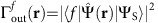, where 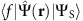 is the QPWF, 
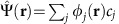
 is the field operator, 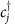 creates a single-hole MO eigenstate *ϕ*_*j*_(**r**) of the system^19^, and the total tunnel rate is 
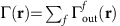
.

From our QPWF description of coupled dopants, we obtain a spatial tunnelling probability 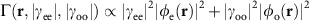 for the ground state. Here, |*γ*_ee_|^2^ and (|*γ*_oo_|^2^) contain constructive (destructive) interference corresponding to even (odd) linear combinations of atomic orbitals *ϕ*_e_(**r**_1_) (*ϕ*_o_(**r**_1_)) (note: |*γ*_ee_|^2^+|*γ*_oo_|^2^=1). To obtain |*γ*_oo_|^2^, data were fit to Γ(**r**, |*γ*_ee_|,|*γ*_oo_|), assuming linear combinations of parametrized *s*-like atomic orbitals for *ϕ*_e_(**r**) and *ϕ*_o_(**r**) appropriate for subsurface acceptors. The QPWF and atomic orbitals are described in Supplementary Figs 4–6.

The least-squares fits in Fig. 3d–f (coloured lines) of Γ(**r**, |*γ*_ee_|, |*γ*_oo_|) are in good agreement with data (squares), for *d*/*a*_B_=2.2, 2.7 and 3.5. For comparison with the data, grey curves are shown for both the uncorrelated (maximally correlated) state with |*γ*_oo_|=0 (|*γ*_oo_|/|*γ*_ee_|=1) in Fig. 3d–f. We note that all three separations exhibit interaction effects at the midpoint of the ions, where the atomic orbital quantum interference is strongest. We obtain |*γ*_oo_|^2^=0.12±0.06, 0.23±0.07 and 0.39±0.08 for *d*/*a*_B_=2.2, 2.7 and 3.5. Data taken at higher tip heights gave identical results to within experimental errors (see Supplementary Figs 7 and 8), independently verifying that the tip does not influence our results.

**Figure 4:**
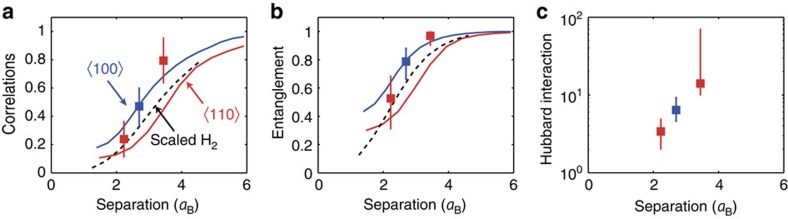
Entanglement entropy and hubbard interactions. (**a**) Quantum correlations versus *d*. Theory predictions are shown for coupled acceptors with 〈110〉 orientations (red line) and 〈100〉 (blue line), alongside scaled H_2_ (dashed black line). Predicted localization is suppressed (enhanced) along 〈110〉 (〈100〉) relative to molecular hydrogen (H_2_), due to valence band anisotropy, which enhances (suppresses) *t*. (**b**) Same as (**a**) for the entanglement entropy 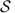. (**c**) Experimentally estimated Hubbard interactions. Error bars denote 95% confidence intervals.

The Coulomb correlations, embodied both in 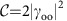 (Fig. 4a) and the entanglement entropy 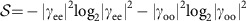 (Fig. 4b), could be evaluated directly from the fit, and both increase with increasing *d*. The one-to-one mapping from 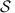 to 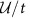 (Fig. 2c) was used to determine the effective Hubbard interactions from the entanglement entropy in Fig. 4b. We obtain 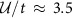, 6.4 and 14, for *d*/*a*_B_=2.2, 2.7 and 3.5, respectively (Fig. 4c), which increase as the tunnel coupling decreases.

We conclude the analysis of the QPWFs with some critical remarks on correlations extracted from our fitting model, recalling that the large spatial overlap of the spectrally overlapping acceptor-bound holes directly shows their states are tunnel coupled. First, the Coulomb correlations have a systematic effect on interference in the QPWF such that the least-squares error is significantly worse if |*γ*_oo_|^2^ is forced to zero in the fitting model (Supplementary Table 1). Second, if applied to very far apart dopants where the ground state can still be resolved, our fitting model would not give a spurious result that the two dopants are highly correlated. This follows because the difference between |*ϕ*_e_(**r**)|^2^ and |*ϕ*_o_(**r**)|^2^, which reflects the interference of atomic orbitals and is used to detect correlations, tends to zero as *d*/*a*_B_ increases. Data (Fig. 3a–c) presented here are for coupled dopants that we found to be (i) well isolated from other dopants or dangling bonds, and (ii) at identical depths, as evidenced by the spatial extent and brightness of the atomic orbitals. When the latter is not satisfied, the atomic levels can be detuned, introducing more parameters to the fit.

### Comparison with theory

These experimental results obey the trends predicted by our theory calculations for the spin-orbit coupled VB. Predictions in Fig. 4a,b for displacements along 〈100〉 (blue solid line) and 〈110〉 (red solid line) both show increasing correlations and entanglement with increasing dopant separation. Moreover, we find that the observed and predicted entanglement entropy qualitatively reproduce a single-band model (Fig. 4a,b, dashed lines). This result implies that inter-hole Hubbard interactions follow an essentially hydrogenic trend with atomic separation, even for non-perturbative interactions 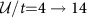.

The hydrogenic nature of 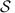 and 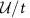 persists in spite of the ± ‘1/2' spin excited states of a single acceptors. Such ±‘1/2' single-acceptor excited states states are found nominally Δ∼1–2 meV above the ±‘3/2' spin ground state due to inversion symmetry breaking at the interface^30^. Although *t*>Δ, 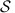 and 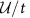 remain hydrogenic in our calculations because the ‘1/2' spin excited state has an *s*-like envelope whose spatial extent is similar to (1) the *s*-like ± ‘3/2' ground state and (2) the scaled hydrogenic ground state. Otherwise, single particle ±‘1/2' states would hybridize stronger than single particle ±‘3/2' states, form the 2-hole singlet at smaller separations, and localize more slowly relative to molecular hydrogen with increasing *d*. Furthermore, the polarization of the ±‘3/2' and ±‘1/2' states into 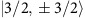 and 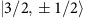 components, respectively, limits the mixing of ±‘1/2' states into the ground state.

### Spin-excited states and effective temperature

**Figure 5:**
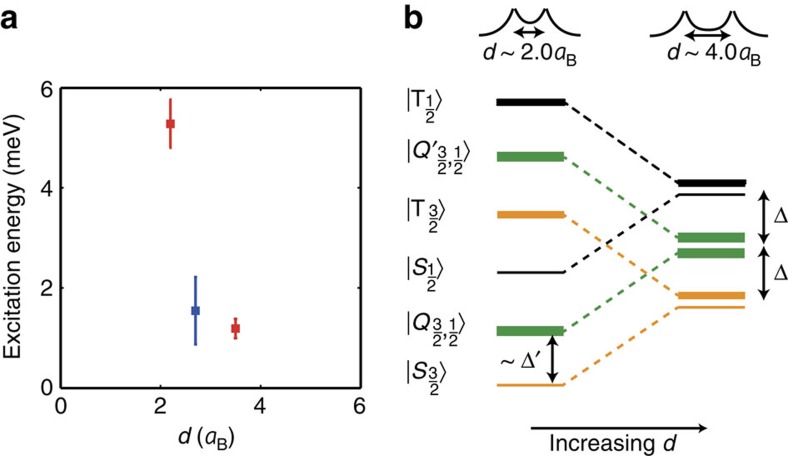
Coupled-spin excitation spectrum. (**a**) Measured energy of first excited state relative to ground state. (**b**) Schematic level diagram of coupled acceptors, reflecting theory calculations, as a function of inter-acceptor distance *d*/*a*_B_. Singlets 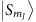 and triplets 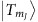 are present for interactions between two holes of *m*_*J*_=±‘3/2' spin (orange) and two holes of *m*_*J*_=±‘1/2' (black) spin. States |*Q*_3/2,1/2_〉 and 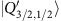 are sets of four closely spaced levels (green) with one ‘3/2' spin hole and one ‘1/2' spin hole. Error bars denote 95% confidence intervals.

Finally, we discuss the observed excited states, which confirm that the inter-acceptor tunnel coupling dominates thermal and tunnel-coupling effects of the reservoir. The energies of the states were determined by fitting the single-hole transport lineshapes^40^ of the coupled acceptors (Supplementary Figs 1 and 2). For the first excited state we found 5.2±0.6 and 1.2±0.2 meV for *d*/*a*_B_=2.2 and 3.5, respectively (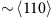 orientation), and 1.6±0.7 meV for *d*/*a*_B_=2.7 (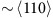 orientation). Shown in Fig. 5a, these energies are too small to add another hole, which would require ≈50 meV for an acceptor in bulk silicon. However, the energies agree well with our predictions for two-hole excited states of coupled hole spins ±‘3/2' and ±‘1/2', that is, 8.5 and 1.5 meV for *d*=2.2*a*_B_ and *d*=3.5*a*_B_ (〈110〉 orientation), and 2.0 meV (〈100〉 orientation). Here we note that some of the predicted coupled-spin excited states (Fig. 5b) are unconventional: a singlet 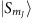 and triplet 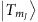 of two ‘3/2' holes (orange lines) and two ‘1/2' holes (black lines) are obtained, where 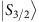 is the ground state for all separations. More subtly, two manifolds 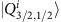, 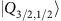, *i*=1 … 4, containing four states are predicted (green lines), where one ±‘3/2' spin level and one ±‘1/2' spin level is occupied. For *d*/*a*_B_=2.2 and 2.7 (*d*/*a*_B_=3.5), the measured energies are in better agreement with predictions for 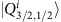 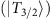 excitations.

The inter-acceptor tunnel couplings *t* (ratios *t*/*T*) were estimated to be 12 meV (30), 7 meV (20) and 3.5 meV (10) for *d*/*a*_B_=2.2, 2.7 and 3.5, respectively, at *T*=4.2 K. Such couplings *t* exceed the reservoir coupling Γ_in_ (Supplementary Table 2) to the substrate by more than 50 × . Combined with bias *U*∼0.2–0.3 V needed to bring the level into resonance, this rules out coherent interactions with substrate and tip reservoirs^41^. Note that the measured energy splittings imply small thermal excited-state populations of ≲10^−5^, ≲10^−2^ and ≲10^−1^ for *d*/*a*_B_=2.2, 2.7 and 3.5, respectively.

## Discussion

We performed atomic resolution measurements resolving spin–spin interactions of interacting dopants, realizing quantum simulation of a two-site Hubbard system. Analyzing these local measurements of the spectral function^17^, we find increasing Coulomb correlations and entanglement entropy as the system is ‘stretched'^23^,^35^,^42^ in the regime of non-perturbative interaction strengths 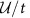. Our experiment is the first to combine low effective temperatures *t*/*T*∼30 at 4.2 K and single-site measurement resolution, considered essential^3^,^5^,^12^ to simulate emergent Hubbard phenomena^9^,^11^. Lower effective temperatures *t*/*T*∼420 are possible at *T*=0.3 K. For example, 4 × 4 Hubbard lattices with 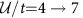 and *t*/*T*∼40 have recently been associated with both the pairing state and pseudogap in systems exhibiting unconventional superconductivity^11^.

The approach generalizes to donors, which can be placed in silicon with atomic-scale precision^27^ and spatially measured *in situ* after epitaxial encapsulation^43^,^44^. In contrast to disordered systems^45^, atomically engineered dopant lattices will require weak coupling to a reservoir, displaced either vertically as demonstrated herein, or a laterally^27^. Strain could be used to further enhance the splitting between light and heavy holes, or suppress valley interference processes of electrons^31^,^46^. Interestingly, open Hubbard systems which may exhibit unusual Kondo behaviour^47^,^48^ could also be studied by this method. The demonstrated measurement of spectral functions could be used to directly determine excitation spectra, evaluate correlation functions^45^ or obtain quasi-particle interference spectra^17^, all of which contain rich information about many-body states, including charge-ordering effects. We envision *in-situ* control of filling factor^9^,^11^, using a back-gate or patterned side-gate^27^. These capabilities will allow for quantum simulation of chains, ladders or lattices^9^,^11^,^49^ at low effective temperatures, having interactions that are engineered atom-by-atom.

## Methods

### Sample preparation

Samples were prepared by flash annealing a boron doped (*p*≈10^19^ cm^−3^) silicon wafer at ∼1,200 °C in ultra-high vacuum (UHV) followed by slow cooling at a rate 1 °C_min_^−1^ to 340 °C. Then, hydrogen passivation was carried out ∼340 °C for 10 min by thermally cracking H_2_ gas at a pressure 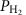
=5 × 10^−7^ mbar.

### Measurements

Atomic resolution single-hole tunnelling spectroscopy was performed at 4.2 K using an UHV Omicron low temperature scanning tunnelling microscope. Current *I* was measured as a function of sample bias *U* and d*I*/d*U* was obtained by numerical differentiation. Details for the analysis of the data are provided in Supplementary Figs 1–3 and 5–8 and Supplementary Notes 1, 2, 4 and 5.

### Theory

Theory calculations of interacting states were carried out using the configuration interaction approach, in the Luttinger–Kohn representation including a realistic description of the heavy-hole (*J*=3/2, |*m*_*J*_|=3/2), light-hole (*J*=3/2, |*m*_*J*_|=1/2) and split-off hole (*J*=1/2, |*m*_*J*_|=1/2) degrees of freedom. Details for the theory are provided in Supplementary Fig. 4 and Supplementary Notes 3, 6 and 7.

## Additional information

**How to cite this article:** Salfi, J. *et al*. Quantum simulation of the Hubbard model with dopant atoms in silicon. *Nat. Commun.* 7:11342 doi: 10.1038/ncomms11342 (2016).

## Supplementary Material

Supplementary Information Supplementary Figures 1-8, Supplementary Tables 1-2, Supplementary Notes 1-7 and Supplementary References
