# Supplementary material for: Quantum simulation of the Hubbard model with dopant atoms in silicon
Source: Nat Commun. 2016 Apr 20;7:11342. doi: 10.1038/ncomms11342 (PMC4842981; doi:10.1038/ncomms11342)
Supplement: Supplementary Information — Supplementary Figures 1-8, Supplementary Tables 1-2, Supplementary Notes 1-7 and Supplementary References [file ncomms11342-s1.pdf]

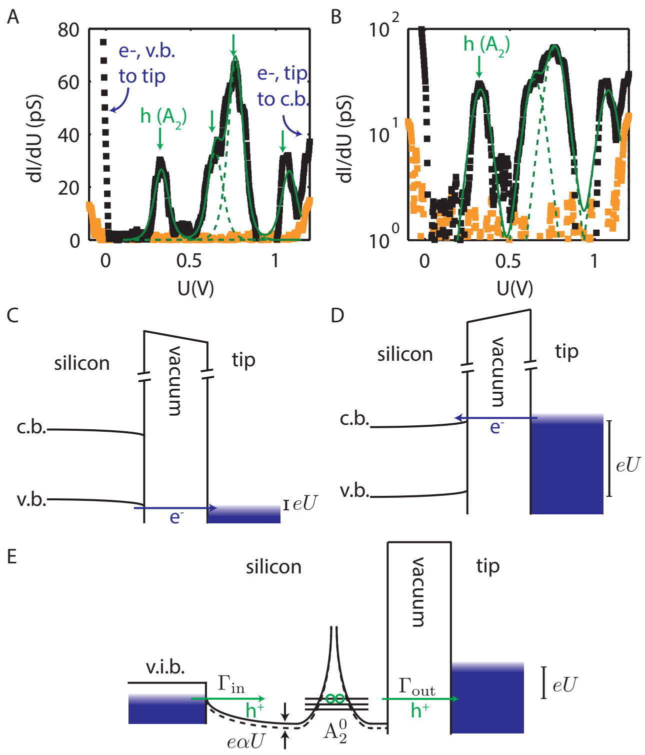

Supplementary Figure 1. A. $\mathrm{d}I/\mathrm{d}U$ vs. U for the $d/a_{\mathrm{B}} = 2.2$, above (black squares) and away from (orange squares) the coupled acceptors. Least square fits (green line) considering sequential hole transport through several peaks (green dashed lines). B. Same as A on logarithmic scale. C. Tunneling of electrons from valence band (v.b.) to the tip. D. Tunneling of electrons from the tip to the conduction band (c.b.). Schematic of hole tunneling at positive sample bias U, from valence impurity band reservoir, to acceptor pair, to tip. Ground and excited states for two-hole occupation are shown. Electrons (holes) in the hole reservoir and STM tip are shaded blue (white).

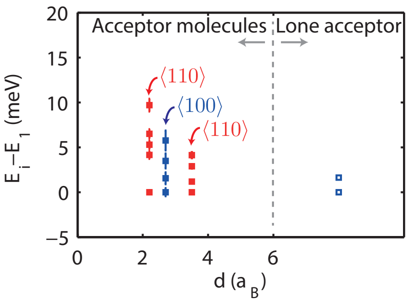

Supplementary Figure 2. Extracted energies $E_i - E_1$ of observed states relative to the lowest energy state (energy E_1), for $d/a_{\mathrm{B}} = 2.2$, $d/a_{\mathrm{B}} = 2.7$, and $d/a_{\mathrm{B}} = 3.5$. For comparison, the energies of the predominantly $\pm$"3/2" and $\pm$"1/2" spin Kramers doublets of a lone acceptor, ~ 1 nm from the Si[001]:H interface, are shown on the right hand side.

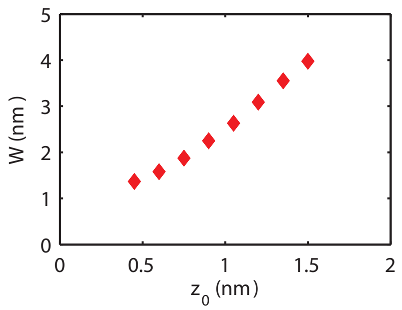

Supplementary Figure 3. Dependence of the full-width half-maximum W of the spatial tunneling probability distribution on depth of acceptor beneath the surface, calculated by numerical diagonalization of the 6×6 spin-orbit coupled Kohn-Luttinger Hamiltonian.

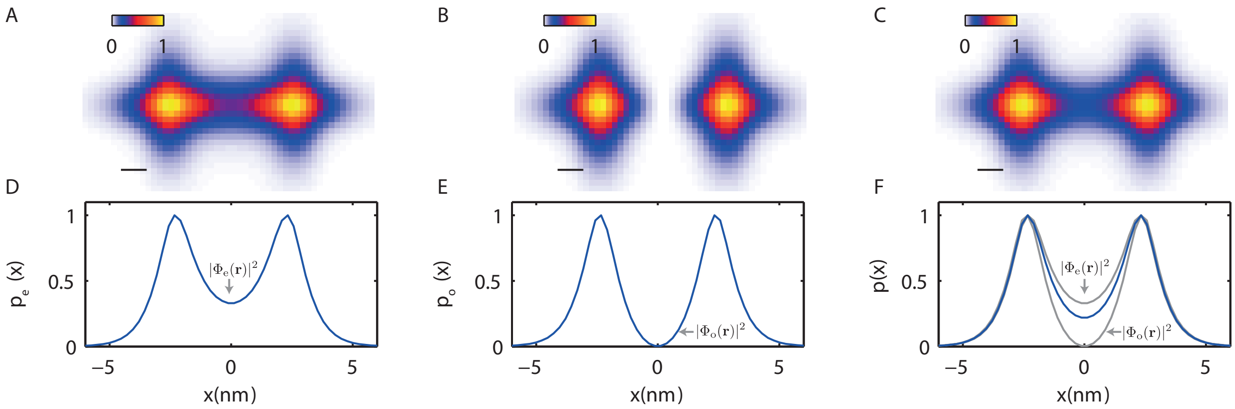

Supplementary Figure 4. A. Predicted spatial tunneling probability distribution for even quasiparticle wavefunctions using 6×6 Kohn-Luttinger. B. Same as A, but for odd quasiparticle wavefunctions. C. Total tunneling probability distribution, a sum of A and B. D. Profile of spatial tunneling probability distribution for even quasiparticle wavefunctions using 6×6 Kohn-Luttinger. E. Same as D, but for odd quasiparticles wavefunctions. F. Total tunneling probability distribution, a sum of of D and E. Scale bar: 1 nm

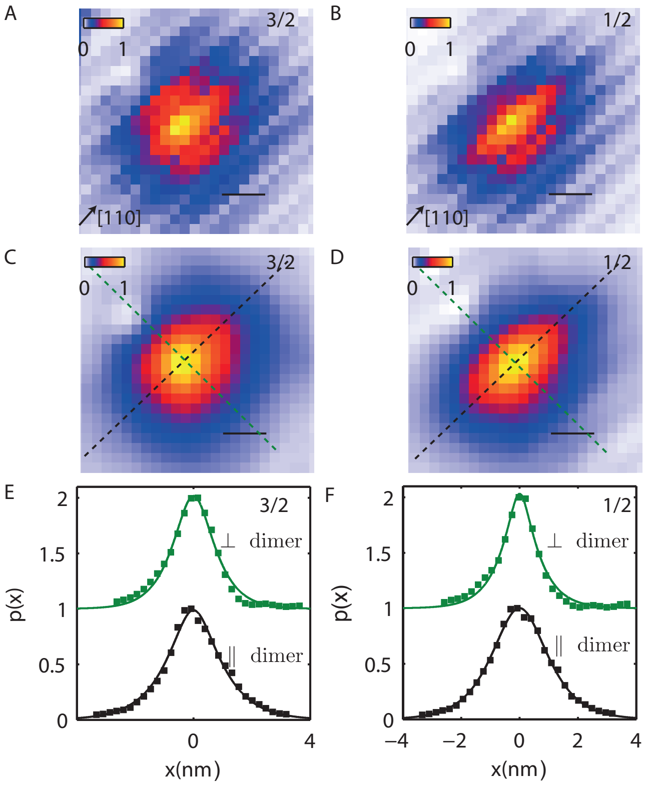

Supplementary Figure 5. A. Integrated dI/dU for single acceptor ground state. B. Integrated dI/dU for first excited state, 1.6 ± 0.4 meV above the ground state. C. Same as A with lattice frequencies filtered out. D. Same as B with lattice frequencies filtered out. E,F. Line profile of relatively tunneling probability parallel (black squares) and perpendicular (green squares, offset for clarity) to the dimer, and fit of profile parallel (black line) and perpendicular (green line, offset for clarity) to dimer direction. F. Same as E, but for first excited state. Scale bar: 1 nm

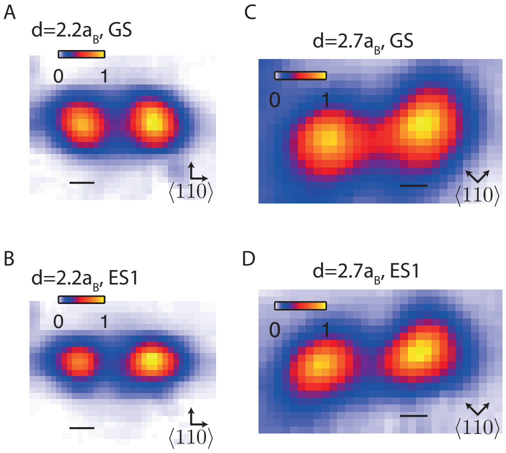

Supplementary Figure 6. A. Spatial measurement of ground state for $d = 2.2a_{\mathrm{B}}$. B. Same as A, but for first excited state. C. Spatial measurement of ground state for $d = 2.7a_{\mathrm{B}}$. D. Same as C, but for first excited state. ⟨110⟩ crystal directions directions are as indicated. Scale bar: 1 nm

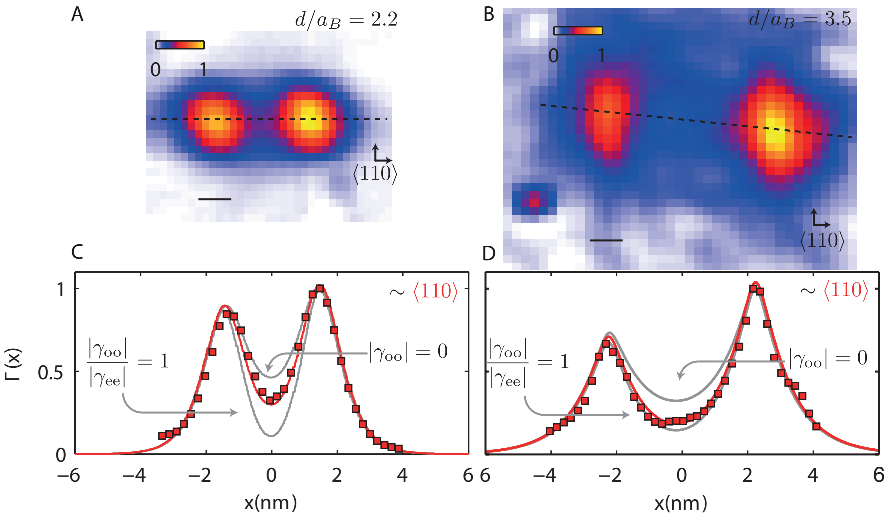

Supplementary Figure 7. A. Experimentally measured, normalized tunneling probability Γ to tip, for $d = 2.2a_{\rm B}$ GS. Arrows denote 110 crystal directions. B. Same as (A), for $d = 3.5a_{\rm B}$. C. Normalized experimental line profile (coloured squares) of $\Gamma(x)$ for $d = 2.2a_{\rm B}$ and least-squares fit (coloured line) to correlated singlet model. Upper and lower grey lines are line profiles of densities $|\phi_e(\mathbf{r})|^2$ and $|\phi_o(\mathbf{r})|^2$ respectively, obtained from least square fits. D. Same as (C), for $d = 3.5a_{\rm B}$. Scale bar: 1 nm

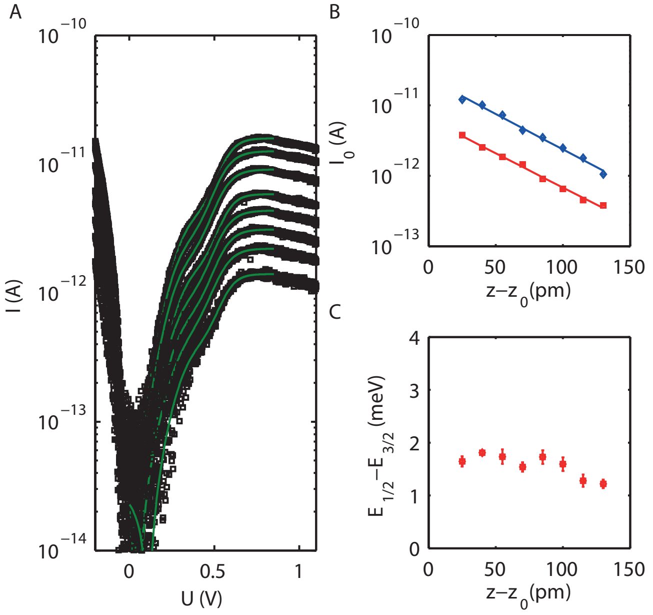

Supplementary Figure 8. A. Tip height dependence of current I vs sample bias U for single acceptor. Two distinct thermally broadened steps corresponding to $\pm 3/2$ states and $\pm 1/2$ states are observed[1]. B. Peak current I_0 reduces exponentially as a function of tip height. C. Energy splitting to the $\pm 1/2$ excited state

d/a_B	Fit # 1		Fit #2	
	$\|\gamma_{oo}\|$	$\overline{SSe}$	$\|\gamma_{oo}\|$	$\overline{SSe}$
2.2	free	0.0159	fixed,0	0.0293
2.7	free	0.0126	fixed,0	0.0509
3.5	free	0.0391	fixed,0	0.0638

Supplementary Table 1. Comparison of sum of square errors when correlation parameter γ_{oo} is a free parameter in fitting model (Fit # 1), versus when γ_{oo} is fixed to zero (Fit #2).

$d(a_B)$	2.2	2.7	3.5
t (GHz)	2900	1700	850
$h\Gamma_\Sigma$ (GHz)	< 2.5	< 30	< 10
$t/h\Gamma_\Sigma$	> 1200	> 60	> 85

Supplementary Table 2. Estimated tunnel coupling $2t$ and environmental coupling rates Γ_Σ for each d. Conservative estimates are given for the molecular coupling, based on calculations for the [100] direction. Nevertheless, even the conservative molecular coupling estimate for the [100] direction well exceed *upper bounds* on the environmental coupling, estimated from the dI/dU lineshape.

Supplementary Note 1. TUNNELING SPECTROSCOPY

In this section we discuss the extraction of the energies of the states observed in dI/dU tunneling spectra. We find that the the number of observed states, the state's relative energies to eachother, and their energies relative to the sample's Fermi energy, are only compatible with two interacting holes on pairs of acceptors. We further estimate the total tunnel coupling Γ_Σ to the tip and sample reservoirs for sequential hole transport, based on the dI/dU lineshape, and show that $h\Gamma_\Sigma$ is much less than the inter-acceptor tunnel coupling t for separations d considered in the main text.

For the acceptor pair with $d/a_B = 2.2$ (Figure 3A, main text), measured dI/dU is plotted in linear scale (Supplementary Figure 1A) and log scale (Supplementary Figure 1B). Away from the acceptor pair (Supplementary Figure 1A/B, orange squares), direct tunneling of electrons from the valence band to the tip (holes from the tip to the valence band) is obtained for $U \lesssim 0$ V as illustrated in Supplementary Figure 1C, while direct tunneling of electrons from the tip to the conduction band occurs for $U \gtrsim 1.15$ V as illustrated in Supplementary Figure 1D. Above the pair (Supplementary Figure 1A/B, black squares), we observe a well-isolated peak at $U \approx 0.3$ V, a collection of two strong (closely spaced) peaks at 0.6 V and 0.75 V, and fourth peak at $U \approx 1.0$ V.

Plotted on a logarithmic scale, both the current (not shown) and numerically differentiated dI/dU data (Supplementary Figure 1B), black squares) grow exponentially with increasing U for the first peak before reaching a local maximum. As suggested by this observation[2,3], we fit the first peak of the differential conductance to a state probed by sequential tunneling with broadening determined by the $T = 4.2$ K temperature of the reservoir, $\cosh^{-2}(e\alpha(U - U_1)/2k_BT)$, where $e = 1.602 \times 10^{-19}$ C is the elementary charge, $k_B = 1.381 \times 10^{-23}$ J/K is the Boltzmann constant, U_1 is the peak voltage, and α is the well-known lever arm from sequential transport[2] describing the variation in the energy level $\Delta E = e\alpha\Delta U$ due to a change in applied bias ΔU, as schematically illustrated in Supplementary Figure 1E.

The least-squares fit is in good agreement with the measured dI/dU for both the full-width at half maximum $\Delta U = 3.6k_BT/e\alpha$ and the exponential decay of the tail $\exp(e\alpha(U - U_1)/k_BT)$. A peak voltage $U_1 = 334.0 \pm 0.9$ meV and a lever arm $\alpha = 0.0144 \pm 0.0006$ were obtained for tunneling of holes from the reservoir, to the acceptor pair, to the tip. The value for α is similar to those found for single-hole transport through individual acceptors in flashed p-type silicon[3] and single arsenic donors in flashed n-type silicon[4]. This excellent agreement shows that broadening of the single-hole transport peaks is due to the temperature associated with the Fermi-Dirac distribution in the hole reservoir.

The small value of α means that the "broadened" lineshape in Supplementary Figure 1A and Supplementary Figure 1B is due to weak electrostatic control of the sample potential by the STM tip, as expected due to strong screening by the carrier reservoir. This is further confirmed by the negligible "spectral shift"[5], that is, the similarity of the apparent gap ≈ 1.15 eV in dI/dU measurements of Supplementary Figure 1A/B to the actual 1.14 eV band gap for heavily-doped p-type silicon at low temperature[6].

A. Energy spectrum

We extracted the lever arm α and energies of states $E_i - E_1 = e\alpha(U_i - U_1)$, by least-squares fitting of dI/dU to a sum of single-hole tunneling peaks[1,3,4,7,8]. Fits

to $\mathrm{d}I/\mathrm{d}U = \sum_i A_i(z)\big(\cosh^{-2}(e\alpha(U-U_i)/2k_BT)\big)$ are found in Supplementary Figure 1A and 1B (solid green lines) along with individual peaks (dashed green lines), on linear and log scales, respectively. Extracted energies $E_i - E_1 = e\alpha(U_i - U_1)$ of the excited states are plotted in Supplementary Figure 2, relative to the energy E_1 of the two-hole ground state, for $d/a_\mathrm{B} = 2.2$, $d/a_\mathrm{B} = 2.7$ and $d/a_\mathrm{B} = 3.5$.

For all d the extracted energy splittings between the states (Supplementary Figure 2) are too small to be associated with single-hole occupied states (A_2^- states) of coupled acceptors. For $d/a_\mathrm{B} = 2.2$, two energetically nearby states were found at 5 and 6.5 meV, while a higher energy state was found at ≈ 3.5 meV above those. Here, the observed splittings are incompatible with the "1s" hybridization energy $2t \approx 25$ meV between tunnel-hybridized single-hole states estimated by numerics (Supplementary Note 3). Similar arguments hold for the $d/a_\mathrm{B} = 2.7$ ($d/a_\mathrm{B} = 3.5$), where the excited state splittings ≈ 2 meV (≈ 1 meV) are incompatible with the single-hole coupling energy scale of $2t \approx 14$ meV ($2t \approx 7$ meV).

Even more importantly, it follows from the electrostatic arguments given below that the coupled-acceptor peaks observed in experiments are too close to the hole reservoir's chemical potential to correspond to ionizations of the very "deep" single-hole (A_2^{-1}) states into zero-hole (A_2^{-2}) states. Rather, they are only compatible with ionizations of the two-hole (A_2^0) ground and excited states of acceptor pairs into one-hole (A_2^{-1}) states, which are energetically very similar to the ionization energy of a neutral (A^0) acceptor. For $d/a_\mathrm{B} = 2.2 \to 3.5$ we found ionization transitions $\mathrm{A}_2^{-1} \to \mathrm{A}_2^{-2}$ for the last hole (Supplementary Note 3) require more than 100 meV energy. This exceeds the Fermi energy $E_F - E_V \approx 50$ meV of the reservoir (E_V is the valence band edge) by more than 50 meV. Now, for $U = 0$ V, the tip's contact potential bends states by an amount $-e\alpha U_\mathrm{FB} = -e\alpha(W - \Phi_\mathrm{tip})$ relative to the sample reservoir, where $W = 5.2$ eV is the work function of degenerately doped p-type reservoir, and Φ_tip is tungsten tip workfunction. Let us assume a work function $\Phi_\mathrm{tip} = 4.8 \pm 0.3$ eV typical of a tungsten tip[3]. Then, the states bend down by 5.8 ± 4.2 meV at zero bias, much smaller than the value > 50 meV required to depopulate a single hole A_2^- state ($\mathrm{A}_2^{-1} \to \mathrm{A}_2^{-2}$ transition). Therefore at zero bias, the observed acceptor pairs are in an A_2^- charge state, and a bias $U = \alpha^{-1} \times 50$ mV much less than -1 V would be required to remove the last hole by tip-induced band bending. This is incompatible with $U \sim 0.2$ to 0.3 V of peaks observed in experiments. On the other hand, ionization energies of the two-hole states, $\mathrm{A}_2^0 \to \mathrm{A}_2^-$ of coupled acceptors are calculated (Supplementary Note 3) to be very energetically similar to A_0 ionization energies. Consequently, transitions populating ground and excited two-hole will occur for $U > 0$, explaining how they (much like single-acceptor ionizing resonances[3]) are observed in our experiments.

B. Transport coupling to reservoir

We have used the $\mathrm{d}I/\mathrm{d}U$ lineshape to obtain an upper bound on the tunnel coupling $\Gamma_\Sigma = \Gamma_\mathrm{in} + \Gamma_\mathrm{out}$ to the environmental reservoirs - the largest lifetime broadening energy scale[2] $h\Gamma_\Sigma$ that can be included in the lineshape analysis of the data[3]. As necessary to observe molecular states, we find Γ_Σ for data in Figure 3A, 3B, and 3C, to be considerably smaller than the inter-acceptor tunnel coupling t estimated from the hybridization energies (Table 2). Tip height-dependent measurements (Supplementary Note 5) confirmed the exponential dependence of the tunnel current on tip height, indicating the rate to the tip is the slower (dominant) rate. The dwell-time (and coupling Γ_Σ), determined by the fast rate, is therefore controlled by the coupling to the hole reservoir in the substrate, Γ_in in Supplementary Figure 1E.

Supplementary Note 2. ACCEPTOR DEPTH ESTIMATE

We have estimated the depth below the silicon surface of the acceptor dopant pairs, presented in Figure 3A, 3B and 3C of the main text, to be approximately 0.5 nm, 0.9 nm, and 0.9 nm, respectively. The depth of isolated dopants in scanning tunneling spectroscopy is typically estimated fitting the spatial variation of a spectral feature such as a band edge, in a bias condition where the dopant is ionized[3,4,9,10], to a dielectric screened Coulomb potential. However, as illustrated in Supplementary Figure 1E, tunneling from the reservoir to the acceptor pair is a transition from a one-hole state to a two-hole state. Consequently, for voltages U below the lowest voltage peak at $U = U_1$ in Supplementary Figure 1A and Supplementary Figure 1B, the two-acceptor system is in Coulomb blockade with single-hole occupation, rather than being doubly ionized, such that fitting to dielectric-screened single-ion potentials is inappropriate[3,4,9,10]. For U above U_1 the two-acceptor system has a high probability of occupation by two holes since $\Gamma_\mathrm{out} \ll \Gamma_\mathrm{in}$. Hence, the method of fitting the band profile to dielectric screened ion potentials used in the above references is problematic.

To estimate the depth of our acceptors, we use the well-known result that the spatial extent of the bound state measured by STS increases with increasing dopant depth (see *e.g.*, reference 11). We compare the full-width at half-maximum W of the spatial tunneling distribution fit in Supplementary Note 4 to the same quantity calculated for subsurface acceptor-bound holes in the 6×6 spin-orbit coupled valence band. We provide this estimate for completeness, since the fitting procedure described in Supplementary Note 4 does not rely on prior knowledge of the depth or Bohr radius of the dopant.

The full-width at half maximum W for the 1s orbital density $\phi_s(\mathbf{r}) \sim \exp(-|\mathbf{r} - \mathbf{r}_0|/a*)$ at $z = 0$ can be directly solved giving $W = 2\sqrt{(\frac{1}{2}a^* \log(\frac{1}{2}) - z_0^*)^2 - (z_0^*)^2}$.

Evaluating this quantity we obtain $W = 1.48 \pm 0.12$ nm, $W = 2.25 \pm 0.39$ nm, and $W = 2.19 \pm 0.25$ nm for the measured molecules with $d/a_{\mathrm{B}} = 2.2$, 2.7, and 3.5, respectively. Comparing these results to the full-width at half maximum W predicted for the orbitals using the numerical solution to Kohn-Luttinger 6×6 Hamiltonian presented in Supplementary Figure 3, the W extracted from experiments translate to depth estimates of ≈ 0.53 nm, ≈ 0.9 nm, and ≈ 0.9 nm, respectively.

A. Correlation with lever arms

When the acceptors are located deeper in the sample, the lever arms are anticipated to be smaller[3]. The relative depths of the planes in which the acceptor pairs are estimated to be located agree with the extracted lever arms. We obtain a lever arm $\alpha = 0.0144 \pm 0.0006$ for the shallower $d/a_{\mathrm{B}} = 2.2$ pair. The other two $d/a_{\mathrm{B}} = 2.7$ and $d/a_{\mathrm{B}} = 3.5$ pairs which are slightly deeper have lever arms $\alpha = 0.0088 \pm 0.0010$ and $\alpha = 0.0055 \pm 0.0003$, respectively. We note that fluctuations in the depth of the reservoir can also influence the lever arm. We anticipate that varying the dopant depths has a small effect on correlations in our experiments where the overall system is neutral. We have observed that the neutral level of near-surface acceptors ($N = 1$) remains bulk-like[3] for depths 0.5-2.0 nm, as expected, due to a competition between the confinement and the dielectric mismatch. Fixed ionization energy essentially fixes the Bohr radius, fixing t and U/t. We note however that the apparent spatial extent of the wavefunction increases with increasing depth. This is not because of a change in the physical Bohr radius. Rather, it occurs because STM probes the wavefunction near the surface.

Supplementary Note 3. THEORY OF INTERACTING ACCEPTORS

We employed a microscopic configuration interaction framework to calculate eigenstates of interacting holes bound to proximate acceptors, in a Kohn-Luttinger 6×6 spin-orbit coupled representation (heavy holes, light holes, and split-off holes), and in a simplified single-band representation for reference. In both representations, the two-hole ground state at relatively small $d/a_{\mathrm{B}} \sim 2$ was found to be predominantly composed of the $|\mathrm{e+e-}\rangle$ singlet of two even orbitals, where $+$ and $-$ denote $+$"3/2" and $-$"3/2" respectively for the valence band holes, and denote $\uparrow$ and $\downarrow$ respectively in the single-band approximation. The probability amplitude of the $|\mathrm{o+o-}\rangle$ singlet of odd orbitals was found to increase with increasing d, signaling interaction-driven generation of quantum correlations. The corresponding spatial tunneling probability distribution was predicted using the quasi-particle wavefunction (QPWF) density appropriate for few-particle states with quantum correlations[12,13], and are presented herein for the non-trivial 6×6 spin-orbit coupled case.

A. Overview

In the absence of spin-orbit coupling, wavefunctions of interacting spins can be classified into singlets and triplets[14]. For holes in the valence band, the atomic orbitals have p-like symmetry, and the angular momentum $L = 1$ couples to the spin (intrinsic) angular momentum $S = 1/2$, such that the resulting Bloch states have definite J and J_z. While intrinsic angular momentum is not a good quantum number, spin can nevertheless be generalized to hole pseudospin, *i.e.*, Kramers doublets of time reversal symmetric linear combinations of Bloch states[15].

Coupling of hole spins is well understood in the Kohn-Luttinger framework[15–17]. It has been theoretically shown that coupled hole pseudospin interact to produce singlets and triplets[15], as for coupled electron spins. In the Kohn-Luttinger approach generalized valence band holes spins as written as spinors $\phi_i(\mathbf{r}) = \sum_{J,m_J} F^i_{J,m_J}(\mathbf{r})|J, m_J\rangle$, where $F^i_{J,m_J}(\mathbf{r})$ are the envelope functions corresponding to the Bloch states $|J, m_J\rangle$. Due to spin-orbit coupling such spinors are ~ 90 % polarized (rather than 100 % polarized) into single Bloch components. This produces a small band-mixing effect when the pseudospins are coupled together[16,17], that we take into account exactly in our numerics.

B. Full-Configuration Interaction

We employ the Kohn-Luttinger FCI approach developed for valence band holes in reference [17]. Two-hole eigenstates $|\Psi\rangle$ are expressed as superpositions of antisymmetrized two-hole Slater determinants $|\alpha\beta\rangle = c^\dagger_\alpha c^\dagger_\beta |0\rangle = 2^{-1/2}(|\alpha\rangle_1 |\beta\rangle_2 - |\beta\rangle_1 |\alpha\rangle_2)$ with probability amplitudes $d_{\alpha\beta}$,

$$|\Psi\rangle = \sum_{\alpha,\beta} d_{\alpha\beta} \, |\alpha\beta\rangle \, . \tag{1}$$

In $|\alpha\beta\rangle$ above, $c^\dagger_j$ creates a single-hole state $|j\rangle = c^\dagger_j|0\rangle$, where $|0\rangle$ is the vacuum state. As discussed in detail below, the single-particle states $|j\rangle$ were chosen as the eigenstates of the non-interacting Hamiltonian $\mathcal{H}_0$ of a pair of acceptor ion potentials and a hard-wall interface with dielectric mismatch. The Hamiltonian for the two-hole system was taken as $\mathcal{H} = \mathcal{H}_0(\mathbf{r}_1) + \mathcal{H}_0(\mathbf{r}_2) + V_{\mathrm{h-h}}(\mathbf{r}_1, \mathbf{r}_2)$ where $V_{\mathrm{h-h}}(\mathbf{r}_1, \mathbf{r}_2)$ is the Coulomb repulsion term for the holes. Substituting $|\Psi\rangle$ above into $\mathcal{H}|\Psi\rangle = E|\Psi\rangle$ and taking the inner product with $\langle\nu\lambda|$, we obtain

$$(E_{0\nu} + E_{0\lambda})d_{\nu\lambda} + J_{\nu\lambda} - K_{\nu\lambda} + \sum_{\alpha\beta} d_{\alpha\beta}(1 - \delta_{\alpha\beta,\nu\lambda})(\Gamma_{\nu\lambda,\alpha\beta} - \Gamma_{\nu\lambda,\beta\alpha}) = E d_{\nu\lambda}, \quad (2)$$

$$\Gamma_{\nu\lambda,\alpha\beta} = \int d\mathbf{r}_1 d\mathbf{r}_2 \Big(\sum_{J,m_j} F^*_{\nu,J,m_j}(\mathbf{r}_1) F_{\alpha,J,m_j}(\mathbf{r}_1) \Big) V_{\mathrm{h-h}}(\mathbf{r}_1, \mathbf{r}_2) \Big(\sum_{J',m'_j} F^*_{\lambda,J',m'_j}(\mathbf{r}_2) F_{\beta,J',m'_j}(\mathbf{r}_2) \Big), \quad (3)$$

for each $|\nu\lambda\rangle$, where $\Gamma_{\nu\lambda,\alpha\beta} = \langle\nu|_1\langle\lambda|_2 V_{\mathrm{h-h}}|\alpha\rangle_1|\beta\rangle_2$ is an off-diagonal (interaction) term evaluated in the spinor representation, $J_{\nu,\lambda} = \Gamma_{\nu\lambda,\nu\lambda}$ is the direct Coulomb interaction, and $K_{\nu\lambda} = \Gamma_{\nu\lambda,\lambda\nu}$ is a direct exchange interaction. Finite $\Gamma_{\nu\lambda,\alpha\beta}$ terms can introduce correlations[17] by creating eigenstates that are admixtures of two-particle Slater determinants $|\alpha\beta\rangle$.

The single particle Hamiltonian employed was $\mathcal{H}_0 = \mathcal{H}(\mathbf{k}) + V_{\mathrm{ion,A}} + V_{\mathrm{ion,B}} + V_{\mathrm{image,A}} + V_{\mathrm{image,B}} + V_{\mathrm{wall}}$ taking into account ion potentials $V_{\mathrm{ion,A/B}}$ of both acceptors, image charges $V_{\mathrm{image,A/B}}$ due to dielectric mismatch[18], and an infinite (hard-wall) V_{wall} potential a distance $1a_{\mathrm{B}} \sim 1$ nm away from the ion representing the semiconductor/vacuum interface. For $\mathcal{H}(\mathbf{k})$ we considered both the 6×6 spin-orbit coupled Kohn-Luttinger valence band[19,20] $\mathcal{H}_{\mathrm{KL}}(\mathbf{k})$, as well as a single-band, isotropic, parabolic dispersion relation $\mathcal{H}_p(\mathbf{k}) = \hbar^2 k^2/2m^*$ where m^* is an effective mass chosen to reproduce boron's binding energy[21]. The latter allows us to calculate states of a simple "scaled hydrogen molecule" without complex spin-orbit coupling and band structure effects. The ion potentials were taken as dielectric-screened Coulomb potentials. For the 6×6 scheme we included additional charge $\delta q = -0.1e$ at the ion site as a crude core-correction to obtain the correct spatial extent $\langle r\rangle$ and ionization energy of the states[22]. The interaction term $V_{\mathrm{h-h}}(\mathbf{r}_1 s, \mathbf{r}_2)$ is a dielectric screened Coulomb interaction including an image charge distribution produced by dielectric mismatch[23].

For the single band theory, the four lowest single-electron eigenstates of $\mathcal{H}_0$ were used to construct 6 possible two-particle configurations, all of which were included for a full configuration interaction (FCI) approach. We considered two even, spin-degenerate orbitals $|\mathrm{e}\sigma\rangle = c^\dagger_{\mathrm{e}\sigma}|0\rangle$, and two odd, spin-degenerate orbitals $|\mathrm{o}\sigma\rangle = c^\dagger_{\mathrm{o}\sigma}|0\rangle$.

For the 6×6 Kohn-Luttinger calculations, we use a basis that reflects the four low-energy states of single acceptors with s-like envelopes - the predominantly $|m_J| = 3/2$ ("3/2") state, and the predominantly $|m_J| = 1/2$ ("1/2") state[24]. Near an interface, the "1/2" state experiences more confinement than the "3/2" state, making the "3/2" state the ground state[1,25,26]. For lone subsurface acceptors ~ 1 nm from an Si[001]:H interface, we have typically measured $\sim 1-2$ meV splittings (see *e.g.*, lone acceptor data in Supplementary Figure 2), in good agreement with few meV values predicted by 6×6 Kohn-Luttinger. See reference [1] for further details. In the two-acceptor potential $\mathcal{H}_0$ these hybridize into eight single-hole eigenstates, which were used to construct 28 possible two-hole configurations, all of which were included in the state vector for a FCI approach. For the acceptor distances in the main text, four were predominantly composed of heavy holes ("3/2" states) and four were predominantly composed of light holes ("1/2" states). In both groups of four states, two are found within a Kramers doublet with even parity symmetry for the majority spin, and two are found within a Kramers doublet having odd parity symmetry for the majority spin[16,17].

A numerical representation of the FCI matrix (Equation 2) was directly diagonalized to obtain state vectors $\mathbf{d}$ and energies E. The FCI matrix was obtained by evaluating the Coulomb matrix elements $J_{\nu\lambda}$, exchange matrix elements $K_{\nu\lambda}$, and the interaction terms $\Gamma_{\nu\lambda,\alpha\beta}$ by Monte-Carlo integration, using the VEGAS method for adaptive sampling and 10^7 iterations per integral. The basis of single particle eigenstates required for the integrals was obtained numerically using the finite difference scheme, for both the single-band and 6×6 representations, on a real-space grid of $110\times110\times110$ points with a discretization step size of 0.21 nm, using Luttinger parameters for silicon's valence band[22]. To normalize the measured and theoretical (6×6 and single-band) inter-acceptor distances, we choose the value $a_{\mathrm{B}} = 1.3$ nm reproducing the 44.4 meV binding energy of a Boron acceptor in silicon[21] in a single-band approximation.

C. Correlations in ground state

In this section, the ground state of the FCI Hamiltonian in Supplementary Note 3 B is discussed. Our FCI predicts a singlet ground state for both the spin-orbit coupled 6×6 representation and for the isotropic single-band case. In a single-band representation (ignoring spin-orbit coupling), the ground state

$$|\Psi_S\rangle = (\gamma_{\mathrm{ee}} c^\dagger_{\mathrm{e},\uparrow} c^\dagger_{\mathrm{e},\downarrow} - \gamma_{\mathrm{oo}} c^\dagger_{\mathrm{o},\uparrow} c^\dagger_{\mathrm{o},\downarrow})|0\rangle \quad (4)$$

was obtained. With increasing d, dynamical interactions (Γ's in Equation 2) resulted in increasing γ_{oo} and increasing quantum correlations $C = 2|\gamma_{\mathrm{oo}}|^2$, as predicted elsewhere[27,28].

The ground state of interacting, spin-orbit coupled holes[16,17] in the 6×6 Kohn-Luttinger representation contains contributions for which majority pseudospin have even parity,

$$\sum_{m_J \neq m'_J} d_{(\mathrm{e},m_J),(\mathrm{e},m'_J)} c^\dagger_{\mathrm{e},m_J} c^\dagger_{\mathrm{e},m'_J} |0\rangle\,, \quad (5)$$

and contributions for which for majority pseudospin have odd parity,

$$\sum_{m_J \neq m'_J} d_{(\mathrm{o},m_J),(\mathrm{o},m'_J)} c^\dagger_{\mathrm{o},m_J} c^\dagger_{\mathrm{o},m'_J} |0\rangle \,. \tag{6}$$

For small $d/a_\mathrm{B} \sim 2$, the dominant configuration (approx. 90 %) in the ground state singlet was found to be $d_{(\mathrm{e},3/2)(\mathrm{e},-3/2)}$, while for increasing d, the probability amplitude $d_{(\mathrm{o},3/2)(\mathrm{o},-3/2)}$ was found to increase. From this, two conclusions can be drawn: (1) the increase in the probability amplitude $d_{(\mathrm{o},3/2)(\mathrm{o},-3/2)}$ with increasing d signals the emergence of hole-hole quantum correlations[17], and (2) the predominance of "3/2" pseudospinspin orbitals signals that the band-mixing effect is weak for the values of d in experiments. Contributions $d_{(\mathrm{e},m_J),(\mathrm{e},m'_J)}$ and $d_{(\mathrm{o},m_J),(\mathrm{o},m'_J)}$ with $m_J = \pm 3/2$ and $m'_J = \pm 3/2$ are much larger than the other "band-mixing" terms with $m_J = \pm$"1/2' or $m'_J = \pm$"1/2".

D. Quasi-particle wavefunction density

In this section, we discuss theoretical predictions of the correlation coefficient C in Figure 4A, as well as theoretical calculation of the quantity measured for spatially resolved single-hole tunneling into multi-particle states with correlations, the QPWF density[12,13,29]. The results of Supplementary Note 3 C are considered, for both the single-band and spin-orbit coupled 6×6 Kohn-Luttinger representations.

As described in Supplementary Note 1, the tunneling probability density measured in experiments is determined by tunneling of a hole from the acceptor molecule to the tip, leaving behind a single-hole eigenstate $|i\rangle$ on the acceptor molecule. The corresponding QPWF density $\Gamma^i_{\mathrm{out}}(\mathbf{r}) \propto |M_{iS}(\mathbf{r})|^2$ is obtained[12], where $M_{iS}(\mathbf{r}) = \langle i|\Psi(\mathbf{r})|\Psi_S\rangle$, $\Psi(\mathbf{r}) = \sum_j c_j \phi_j(\mathbf{r})$ is the field operator, $\mathbf{r} = (x, y, z_0)$ is position of the tip apex orbital, and $|\Psi_S\rangle$ is the ground state. For the single-hole transport processes probed, it is necessary to sum over the final (single-hole) states $|i\rangle$ of the molecule after hole tunneling to the tip. The total tunneling probability density is then $\Gamma_{\mathrm{out}}(\mathbf{r}) = \sum_i \Gamma^i_{\mathrm{out}}(\mathbf{r})$.

For the single band case, evaluating the QPWF using the field operator gives $\Gamma_{\mathrm{out}}(\mathbf{r}) \propto \left[|\gamma_{ee}|^2|\phi_e(\mathbf{r})|^2 + |\gamma_{oo}|^2|\phi_o(\mathbf{r})|^2\right]$. Results for $C = 2|\gamma_{oo}|^2$ are plotted in the main text (Figure 4A, dashed line).

For the 6×6 spin-orbit coupled representation, the quasi-particle wavefunction densities are also readily obtained, such that $\Gamma_{\mathrm{out}}(\mathbf{r}) = \sum_i |M_{iS}(\mathbf{r})|^2$, where $M_{iS}(\mathbf{r}) = \sum_{\alpha\beta} d_{\alpha\beta}[\phi_\alpha(\mathbf{r})\delta_{i\beta} - \phi_b(\mathbf{r})\delta_{i\alpha}]$. Evaluating this expression results in a QPWF density that is a sum of even and odd QPWF contributions for all d and both orientations $\langle 100\rangle$ and $\langle 110\rangle$, as expected considering the generalized parity of holes[16]. Results for C, again defined as twice the probability density of odd orbitals, are plotted in the main text (Figure 4A, coloured lines) for both $\langle 100\rangle$ and $\langle 110\rangle$ orientations.

The predicted spatial tunneling probability density is shown 1 nm above the ion in Supplementary Figure 4 for an acceptor molecule with $d = 3.8a_\mathrm{B}$. The predicted correlation coefficient is $C = 0.6$, similar to $C = 0.78 \pm 0.16$ extracted from measurements of the $d = 3.5a_\mathrm{B}$ acceptor molecule, in Figure 4A of the main text. Contributions to the spatial maps (line profiles) from even and odd QPWF densities are separately plotted in Supplementary Figure 4A (Supplementary Figure 4D) and Supplementary Figure 4B (Supplementary Figure 4E), respectively. Their sum, the total tunneling probability density (line profile), is plotted in Supplementary Figure 4C (Supplementary Figure 4F). Upper and lower grey lines in Supplementary Figure 4F denote normalized QPWF densities for the even QPWF and odd QPWF components, respectively, which in this case directly correspond to the profiles in Supplementary Figure 4D and 4E, respectively.

Supplementary Note 4. FITTING OF MEASURED SPATIAL TUNNELLING PROBABILITIES

The experimentally obtained spatial tunneling probabilities for the two-hole ground state of acceptors, in Figure 3A, Figure 3B, and Figure 3C of the main text, were fit assuming even and odd linear combinations of atomic orbitals with s-like envelopes for $\phi_e(\mathbf{r})$ and $\phi_o(\mathbf{r})$. This data was obtained using a tip height established by constant current imaging conditions, at a bias where the topography was nominally flat apart from dimer row corrugations.

In this section we describe the fits and show that the probability density of an s-like wavefunction describes the measured probability density of the ground state and first excited state of an isolated acceptor, in agreement with what is expected for the s-like "3/2" pseudospin ground state and s-like "1/2" pseudospin excited state.

Spatially resolved tunneling spectra were measured for isolated acceptors, consistently revealing two $\mathrm{d}I/\mathrm{d}U$ peaks associated with the s-like $J = 3/2$ ground state manifold. The ~ 2 meV splitting of the isolated acceptor in Supplementary Figure 2 was found to be in good agreement with single-particle eigenstates calculated from our numerical Kohn Luttinger solver for depths determined as in previous work[3], that is, by a fitting the profile to the ionized acceptor's perturbation to the band edge[9,10]. The spatial tunneling probability distributions for the lowest energy state and first excited state of an isolated acceptor were obtained by integrating the two low energy $\mathrm{d}I/\mathrm{d}U$ peaks. Results are shown in Supplementary Figure 5A and Supplementary Figure 5B for the single acceptor in Supplementary Figure 2.

The dimer lattice structure of the hydrogen-terminated 2×1 surface is evident in the states Supplementary Figure 5A and Supplementary Figure 5B, running along the [110] direction indicated. The dimer and lattice frequencies were Fourier filtered out of the image[30], after which

only the characteristic envelope of the probability density of the states remain. Results for the ground state and first excited state are presented in Supplementary Figure 5C and 5D, respectively. The tunneling probability density reflects the hole density, and the appearance of two s-like envelopes is in agreement with expectations of the s-like envelopes for the "3/2" and "1/2" states[1,24]. We observe that the measured low-frequency envelope for the probability density is closer to isotropic for the ground state, and spatially elongated along the dimer direction for the excited state.

The probability density was fit along a line in the plane ($z = 0$) of the surface of the form $|\phi_s(\mathbf{r})|^2$, where $\phi_s(\mathbf{r}) \sim \exp(-|\mathbf{r} - \mathbf{r}_0|/a*)$ is a 1s orbital envelope function. In this expression, $\mathbf{r} = (x, y, z)$ is the tip position, $\mathbf{r}_0 = (x_0, y_0, z_0*)$, x_0 and y_0 are centre coordinates of the orbital, z_0^* is an effective depth, and a^* is an effective Bohr radius. The profile of the measured ground state (solid squares) and least-square fit (solid line) are shown for a profile along the direction parallel (black) and perpendicular (green) to the dimer row in Supplementary Figure 5E, demonstrating excellent agreement. The same quantities are plotted for the first excited state in Supplementary Figure 5F. A more noticeable anisotropy of the excited "1/2" state was found.

Motivated by the theoretical observation that the QPWF density partitions into contributions from even and odd states, we categorize the measured QPWF density into contributions from even and odd QPWFs, that is, linear combinations of atomic orbitals with even and odd parity, respectively. For a distance R between acceptors, we use $\phi_e(\mathbf{r}) = (1/I_e(R))[\phi_s(\mathbf{r}-\mathbf{R}/2)+\phi_s(\mathbf{r}+\mathbf{R}/2)]$ and $\phi_o(\mathbf{r}) = (1/I_o(R))[\phi_s(\mathbf{r} - \mathbf{R}/2) - \phi_s(\mathbf{r} + \mathbf{R}/2)]$, where $I_e(r) = 1 + (1 + r + r^2/3)\exp(-r)$ and $I_o(r) = 1 - (1 + r + r^2/3)\exp(-r)$ in atomic units. In the fits presented in the main text, a^*, z_0^*, $\mathbf{R}$, and $|\gamma_{ee}|^2$ are free parameters, while $1 = |\gamma_{oo}|^2 + |\gamma_{ee}|^2$ is simply a consequence of normalization. Effective Bohr radii $a^* \sim 1$ nm and depths $z_0^* \sim 1$ nm were obtained from the least squares fits. In our fits the values for a^* and z_0^* define a full-width at half maximum W for the spatial tunneling probability of each acceptor. In Supplementary Note 2, the actual depth z_0 of the acceptors was estimated using W derived from experimentally fit values a^*, z_0^*.

A. Error analysis in fit

The sum of square errors

$$\overline{SSe} = \sum_{x_i} (\Gamma(x_i) - \Gamma_{\text{fit}}(x_i))^2/(\Gamma(x_i))^2 \quad (7)$$

was used to determine the importance of the Coulomb correlation parameter γ_{oo} in the fits. The first column (Fig #1) of Supplementary Table 1 gives the results for the $\overline{SSe}$ when $|\gamma_{\text{oo}}$ is a free parameter, while the second column (Fig # 2) of Supplementary Table 1 gives the results for $\overline{SSe}$ when $|\gamma_{\text{oo}}$ is artificially forced to zero. Here we see the fit is considerably improved by including the Coulomb correlation parameter in the fit.

B. Independence of fit results on tip height

Due to the very small lever arm $\alpha \sim 10^{-2}$, the data is acquired very near flat-band[3], and as such we do not observe the so-called "ionization parabolas" (observed elsewhere and associated with tip-induced band bending[9,31]) for single B:Si acceptors[1], or for B:Si acceptor pairs (Fig. 1b, main text).

To provide an independent check that the tip does not influence the results of the QPWF measurement, we present measurements on the $d/a_{\text{B}} = 2.2$ and $d/a_{\text{B}} = 3.5$ pairs from the main text at a tip height +60 pm above the position where the data was taken in the main text. The normalized tunneling probability is shown in Supplementary Fig 7A for $d/a_{\text{B}} = 2.2$ and in Supplementary Fig 7B for $d/a_{\text{B}} = 3.5$. Line profiles of the normalized tunneling probability (squares, Supplementary Fig 7C and Supplementary Fig 7D), are in excellent agreement with the same QPWF model (solid red line, Supplementary Fig 7C and Supplementary Fig 7D) used in the main text. Reference curves are shown for the totally uncorrelated state ($\gamma_{\text{oo}} = 0$) and maximally correlated state ($\gamma_{\text{oo}}/\gamma_{\text{ee}} = 1$).

The extracted results for the correlations are $|\gamma_{\text{oo}}|^2 = 0.15 \pm 0.06$ for $d/a_{\text{B}} = 2.2$ and $|\gamma_{\text{oo}}|^2 = 0.40 \pm 0.09$ for $d/a_{\text{B}} = 3.5$. These results for +60 pm tip heights are within the experimental errors of the results for the data in the main text, which are $|\gamma_{\text{oo}}|^2 = 0.12 \pm 0.06$ for $d/a_{\text{B}} = 2.2$ and $|\gamma_{\text{oo}}|^2 = 0.39 \pm 0.08$ for $d/a_{\text{B}} = 3.5$.

C. Large distance limit of fitting model

For any given experimental signal-to-noise ratio, there always exists a large enough d/a_{B} where it is impossible to distinguish the existence of correlations in the ground state QPWF. This follows from the fact that our model determines correlations based on the contrast between orbitals $|\phi_{\text{e}}(\mathbf{r})|^2$ and $|\phi_{\text{o}}(\mathbf{r})|^2$. However, since $|\phi_{\text{e}}(\mathbf{r})|^2 - |\phi_{\text{o}}(\mathbf{r})|^2 \sim 2\phi_A(\mathbf{r})\phi_B(\mathbf{r})$, where $\phi_{A/B}(\mathbf{r})$ are atomic orbitals at sites A and B, and the product $2\phi_A(\mathbf{r})\phi_B(\mathbf{r})$ is exponentially suppressed with increasing d/a_{B}. The results of Table 1 show that the Coulomb correlations embodied by $|\gamma_{\text{oo}}|$ can be reliably detected, for the signal-to-noise ratio of our experiment.

D. Spatial measurements of excited states

We present the ground and excited states of the acceptor pairs for $d/a_{\text{B}} = 2.2$ and 2.7 in Supplementary Figure 6. Compared with the ground states for $d/a_{\text{B}} = 2.2$ and $d/a_{\text{B}} = 2.7$ in Supplementary Figure 6A and Supplementary Figure 6C respectively, the excited states for

$d = 2.2a_{\rm B}$ (Supplementary Figure 6B) and $d = 2.7a_{\rm B}$ (Supplementary Figure 6D) are slightly spatially elongated along the 110 direction. This is consistent with the expected QPWF density in the interpretation of Figure 5 (main text), that the excited state orbitals have one hole occupying a "1/2" pseudospin state and the other occupying a "3/2" pseudospin state. These states display slightly different spatial anisotropies (Supplementary Figure 5C and Supplementary Figure 5D).

While we have measured the spatial tunneling probabilities for the excited states, measuring excited-state quasi-particle wavefunctions requires slow tunnel-in from the tip into the dopant system (not slow tunnel-out to the tip). Slow tunnel-in from the tip is required to make the current limiting slow tunnel rates additive for excited states (rather than competitive with the ground state as in the case herein), in the presence of Coulomb blockade. Some details are given in reference 8.

Supplementary Note 5. TIP-HEIGHT DEPENDENT SPECTRA

In this section we discuss our confirmation of our model for the thermally broadened sequential tunneling in Supplementary Fig 1E, by detailed investigation of the tip-height dependence of the current, in agreement with our results on Arsenic donors in Si[4,8]. This is shown in Supplementary Figure 8A for tip heights that sequentially increase by 15 pm per curve, above a single B:Si acceptor presenting the unique topographical signature discussed elsewhere[1,3]. The data shows broadened current steps centred at $U \approx 0.25$ V and $U \approx 0.5$ V that have an exponential dependence on the tip height, and which fit very well the lineshape of purely thermally broadened current resonance of a dopant[1,3,4,7,8]. The peaks shown here correspond to the $\pm 3/2$ ground state and $\pm 1/2$ first excited state discussed in Supplementary Note 4 and reference 1.

Qualitatively, the exponential dependence on tip height verifies the assumption that $\Gamma_{\rm in} \gg \Gamma_{\rm out}$, as expected for the large vacuum tunneling barrier, and as required to obtain the results in Figure 3 of the main text. We performed least-squares fitting to extract the height I_0 of the thermally broadened current steps. These I_0 are plotted as a function of tip height $z - z_0$ in Supplementary Figure 8b for the $\pm 3/2$ ground state (red curve) and the $\pm 1/2$ excited state (blue curve). The data are in excellent agreement with a z-dependence $\exp(-2\kappa(z - z_0))$ where $\kappa = (1.15 \pm 0.05) \times 10^{10}$ m^{-1}. This value is in good agreement with the expected value for $\kappa = \sqrt{2m_0\Phi_B}/\hbar$ for the ≈ 5 eV barrier expected for shallow valence band acceptor in silicon. Moreover, we find that the lever arm (not shown) and energy splitting (Supplementary Figure 8C) between the ground and excited state does is essentially independent of tip height. This provides further verification of the sequential tunneling model, and that the tip does not strongly influence the acceptor-bound states.

Supplementary Note 6. HUBBARD MODEL VS. MOLECULAR ORBITALS

The results for the two-site Hubbard model and its relationship to the molecular orbital model in Figure 2b of the main text are straightforward to derive. The coefficients $\gamma_{\rm c}$ and $\gamma_{\rm i}$ of the singlet ground state $|\Psi_S\rangle = \gamma_{\rm c}(|\uparrow;\downarrow\rangle - |\downarrow;\uparrow\rangle) + \gamma_{\rm i}(|\uparrow\downarrow;\rangle + |;\uparrow\downarrow\rangle)$ (Figure 2b, main text) are readily found by direct diagonalization to be $\gamma_c = a/\sqrt{2+a^2}$ and $\gamma_i = 1/\sqrt{2+2a^2}$ where $a = (-U/4t + \sqrt{1+(U/4t)^2})^{-1/2}$.

The dependence of γ_{ee} and $\gamma_{\rm oo}$ on U/t in Figure 2b can be found by a simple transformation. The orthonormal localized states of the Hubbard dimer model created by operators $c^\dagger_{A\sigma}$ and $c^\dagger_{B\sigma}$ are

$$c^\dagger_{A\sigma} = w^{-1/2}(c^\dagger_{a\sigma} - g c^\dagger_{b\sigma}) \text{ and} \tag{8}$$

$$c^\dagger_{B\sigma} = w^{-1/2}(c^\dagger_{b\sigma} - g c^\dagger_{a\sigma}) \tag{9}$$

respectively[32]. Here $c^\dagger_{a\sigma}$ and $c^\dagger_{b\sigma}$ create (non-orthogonal) atomic orbitals with spin σ, $w = 1 - 2Sg + g^2$, $g = (1 - \sqrt{1-S^2})/S$, and $S = \langle a|b\rangle$ is the overlap between the atomic orbitals.

Re-writing the even and odd eigenstates of the single-particle potential and equation the two singlet ground states we find $\gamma_{\rm ee} = \gamma_{\rm c} + \gamma_{\rm i}$ and $\gamma_{\rm oo} = \gamma_{\rm c} - \gamma_{\rm i}$. These relationships are used to map the Hubbard model solution to the molecular orbital model in Figure 2B of the main text. This mapping is independent of normalization constants w, g, and S.

Supplementary Note 7. DEGREE OF ENTANGLEMENT

For fermions, entanglement is defined in terms of Slater decompositions[33] rather than the Schmidt decompositions of distinguishable particles, and can be expressed independent of basis by a degree of entanglement. Here we follow derivation in reference [28] for entanglement to describe correlations in a two-site Fermi-Hubbard system. For two particles that obey Fermi statistics,

$$|\Psi\rangle = \sum_{a,b} \omega_{a,b} |a\rangle_1 |b\rangle_2 \tag{10}$$

is characterized by an anti-symmetric matrix $\omega_{a,b}$, that is, a Slater decomposition. Transformed into a block diagonalized form diag$[Z_0, Z_1, ..., Z_N]$ through a unitary rotation of the single particle states, where

$$Z_i = \begin{pmatrix} 0 & z_i \\ -z_i & 0 \end{pmatrix}, \tag{11}$$

the number of nonzero z_i is called the Slater rank, and if the Slater rank is 1, the quantum correlation of the state is zero[34]. It has been shown[28] that the z_i^2 are the

eigenvalues of the basis independent quantity $\omega^\dagger\omega$, and based on this observation, the degree of entanglement $\mathcal{S}$ of the two particles was defined as

$$\mathcal{S} = -\sum_i z_i^2 \log_2 z_i^2. \qquad (12)$$

For a superposition of "even/even" and "odd/odd" singlets $|S\rangle = (\gamma_{\rm ee} c^\dagger_{\rm e,\uparrow} c^\dagger_{\rm e,\downarrow} - \gamma_{\rm oo} c^\dagger_{\rm o,\uparrow} c^\dagger_{\rm o,\downarrow})|0\rangle$,

$$\omega = \begin{pmatrix} 0 & \gamma_{\rm ee} & 0 & 0 \\ -\gamma_{\rm ee} & 0 & 0 & 0 \\ 0 & 0 & 0 & -\gamma_{\rm oo} \\ 0 & 0 & \gamma_{\rm oo} & 0 \end{pmatrix}. \qquad (13)$$

It directly follows that the non-zero eigenvalues z_i^2 of $\omega^\dagger\omega$ are $|\gamma_{\rm ee}|^2$ and $|\gamma_{\rm oo}|^2$. Then, the degree of entanglement for the state $|S\rangle$ is

$$\mathcal{S} = -|\gamma_{\rm ee}|^2 \log_2 |\gamma_{\rm ee}|^2 - |\gamma_{\rm oo}|^2 \log_2 |\gamma_{\rm oo}|^2. \qquad (14)$$

8. SUPPLEMENTARY REFERENCES

---

[1] Mol, J. A. et al. Interface-induced heavy-hole/light-hole splitting of acceptors in silicon **106**, 203110 (2015).

[2] Foxman, E. et al. Effects of quantum levels on transport through a Coulomb island. Phys. Rev. B **47**, 10020–10023 (1993).

[3] Mol, J. A., Salfi, J., Miwa, J. A., Simmons, M. Y. & Rogge, S. Interplay between quantum confinement and dielectric mismatch for ultrashallow dopants. Phys. Rev. B **87**, 245417 (2013).

[4] Salfi, J. et al. Spatially resolving valley quantum interference of a donor in silicon. Nature Materials **13**, 605–610 (2014).

[5] Lin, H.-A., Jaccodine, R. J. & Freund, M. S. Elimination of spectral shifts associated with tip-induced band bending in scanning tunneling spectroscopy of lightly doped silicon **73**, 2462 (1998).

[6] Wagner, J. & del Alamo, J. A. Band-gap narrowing in heavily doped silicon: A comparison of optical and electrical data. Journal of Applied Physics **63**, 425–429 (1988).

[7] van der Heijden, J. et al. Probing the Spin States of a Single Acceptor Atom. Nano Lett. **14**, 1492–1496 (2014).

[8] Voisin, B., Salfi, J., Bocquel, J., Rahman, R. & Rogge, S. Spatially resolved resonant tunneling on single atoms in silicon. J. Phys.: Condens. Matter **27**, 154203 (2015).

[9] Teichmann, K. et al. Controlled Charge Switching on a Single Donor with a Scanning Tunneling Microscope. Phys. Rev. Lett. **101**, 076103 (2008).

[10] Lee, D.-H. & Gupta, J. A. Tunable Field Control Over the Binding Energy of Single Dopants by a Charged Vacancy in GaAs. Science **330**, 1807–1810 (2010).

[11] Çelebi, C. et al. Surface Induced Asymmetry of Acceptor Wave Functions. Phys. Rev. Lett. **104**, 086404 (2010).

[12] Rontani, M. & Molinari, E. Imaging quasiparticle wave functions in quantum dots via tunneling spectroscopy. Phys. Rev. B **71**, 233106 (2005).

[13] Maruccio, G. et al. Correlation Effects in Wave Function Mapping of Molecular Beam Epitaxy Grown Quantum Dots. Nano Lett. **7**, 2701–2706 (2007).

[14] Mattis, D. C. Theory of magnetism, vol. 17 (Springer Series in Solid-State Sciences, 1981).

[15] Kavokin, K. Symmetry of anisotropic exchange interactions in semiconductor nanostructures. Phys. Rev. B **69**, 075302 (2004).

[16] Climente, J., Korkusinski, M., Goldoni, G. & Hawrylak, P. Theory of valence-band holes as Luttinger spinors in vertically coupled quantum dots. Phys. Rev. B **78**, 115323 (2008).

[17] Yakimov, A. I., Bloshkin, A. A. & Dvurechenskii, A. V. Calculating the energy spectrum and electronic structure of two holes in a pair of strained Ge/Si coupled quantum dots. Phys. Rev. B **81**, 115434 (2010).

[18] Hao, Y., Djotyan, A., Avetisyan, A. & Peeters, F. Shallow donor states near a semiconductor-insulator-metal interface. Phys. Rev. B **80**, 035329 (2009).

[19] Luttinger, J. & Kohn, W. Motion of Electrons and Holes in Perturbed Periodic Fields. Phys. Rev. **97**, 869–883 (1955).

[20] Chao, C. & Chuang, S. Spin-orbit-coupling effects on the valence-band structure of strained semiconductor quantum wells. Phys. Rev. B **46**, 4110–4122 (1992).

[21] Belyakov, V. A. & Burdov, V. A. Anomalous splitting of the hole states in silicon quantum dots with shallow acceptors. J. Phys.: Condens. Matter **20**, 025213 (2007).

[22] Bernholc, J. & Pantelides, S. Theory of binding energies of acceptors in semiconductors. Phys. Rev. B **15**, 4935–4947 (1977).

[23] Orlandi, A., Rontani, M., Goldoni, G., Manghi, F. & Molinari, E. Single-electron charging in quantum dots with large dielectric mismatch. Phys. Rev. B **63**, 045310 (2001).

[24] Bir, G. L., Butikov, E. I. & Pikus, G. E. Spin and combined resonance on acceptor centres in Ge and Si type crystals—II. Surface Science **24**, 1475–1486 (1963).

[25] Gammon, D., Merlin, R., Masselink, W. & Morkoc, H. Raman spectra of shallow acceptors in quantum-well structures. Phys. Rev. B **33**, 2919–2922 (1986).
[26] Masselink, W., Chang, Y.-C. & Morkoc, H. Acceptor spectra of AlGaAs-GaAs quantum wells in external fields: Electric, magnetic, and uniaxial stress. Phys. Rev. B **32**, 5190–5201 (1985).
[27] Coulson, C. A. & Fischer, I. Notes on the molecular orbital treatment of the hydrogen molecule. Philosophical Magazine **40**, 383–393 (1949).
[28] He, L., Bester, G. & Zunger, A. Singlet-triplet splitting, correlation, and entanglement of two electrons in quantum dot molecules. Phys. Rev. B **72**, 195307 (2005).
[29] Schulz, F. et al. Many-body transitions in a single molecule visualized by scanning tunnelling microscopy. Nature Physics (2015).
[30] Çelebi, C. et al. Anisotropic spatial structure of deep acceptor states in GaAs and GaP. Phys. Rev. B **77**, 075328 (2008).
[31] Teichmann, K. et al. Bistable Charge Configuration of Donor Systems near the GaAs(110) Surfaces. Nano Lett. **11**, 3538–3542 (2011).
[32] Schliemann, J., Loss, D. & MacDonald, A. Double-occupancy errors, adiabaticity, and entanglement of spin qubits in quantum dots. Phys. Rev. B **63**, 085311 (2001).
[33] Amico, L., Osterloh, A. & Vedral, V. Entanglement in many-body systems. Rev. Mod. Phys. **80**, 517–576 (2008).
[34] Schliemann, J., Cirac, J., Kuś, M., Lewenstein, M. & Loss, D. Quantum correlations in two-fermion systems. Phys. Rev. A **64**, 022303 (2001).
